# Suitability of Improved and Ancient Italian Wheat for Bread-Making: A Holistic Approach

**DOI:** 10.3390/life12101613

**Published:** 2022-10-15

**Authors:** Marina Mefleh, Fatma Boukid, Costantino Fadda

**Affiliations:** 1 Department of Agronomy, University of Sassari, Via De Nicola, 07100 Sassari, Italy; 2 ClonBio Group Ltd., 6 Fitzwilliam Pl, D02 XE61 Dublin, Ireland

**Keywords:** ancient wheat, genetic improvement, grain composition, dough rheology, bread

## Abstract

Ancient and old wheat grains are gaining interest as a genetic reservoir to develop improved Italian genotypes with peculiar features. In this light, the aim of this study was to assess the baking performance of two improved einkorn (Monlis and Norberto) and two improved emmer (Padre Pio and Giovanni Paolo) genotypes in comparison with two Italian landraces (Garfagnana and Cappelli) and Khorasan. This set was evaluated following a holistic approach considering the flour, dough, and bread properties. The results showed that the flour properties, dough rheology, pasting, and fermentation parameters, as well as the bread properties, significantly differed among the studied genotypes. Cappelli produced the bread with the best quality, i.e., the highest volume and lowest firmness. Despite having the same pedigrees, Giovanni Paolo and Padre Pio resulted in significantly different technological properties. Giovanni Paolo flour showed the highest protein content and provided a dough with a high gas production capacity, resulting in the bread having a similar firmness to Cappelli. Padre Pio flour provided bread having a similar volume to Cappelli but a high firmness similar to Khorasan and Garfagnana. The einkorn genotypes, Monlis and Norberto, showed poor fermentation properties and high gelatinization viscosity that resulted in bread with poor quality. Alternatively, they could be more suitable for making non-fermented flatbreads. Our results showed that the improved wheat showed a high versatility of features, which offers bakers a flexible material to make a genotype of bread types.

## 1. Introduction

Ancient wheat species, einkorn (*Triticum monococcum* ssp. *monococcum*), emmer (*Triticum turgidum* ssp. *dicoccum*), and spelt (*Triticum aestivum* subsp. *spelt*), can be defined as populations of primitive grains that were not subject to any breeding or selection, and thus retained their wild characteristics, such as ear height, a brittle rachis, and a low harvest index [1]. The domestication of emmer originated old durum (*Triticum turgidum* ssp. *durum* Desf.), turanicum (*Triticum turgidum* ssp. *turanicum*) and other tetraploid wheat grains [2]. Since 1960, a drastic replacement of old genotypes with modern ones was driven with the aim of developing high-yielding genotypes with strong gluten suitable for pasta-making. 

In recent years, the cultivation of ancient and old wheat grains has been on an upward trend for several motives. Breeders are reusing the heterogeneous ancient grains, landraces, and old genotypes to exploit them as a source of genetic diversity for the identification of favorable alleles and to preserve natural agrobiodiversity [1,3,4,5]. Ancient grains are also known for their high adaptability to harsh conditions, such as marginal areas, low agronomic input, and organic farming [2,6,7]. These grains were reported to be sources of proteins, minerals, carotenoids, and polyphenols [8,9,10]. Their high nutritional quality and their content of health-beneficial compounds make them extremely attractive for bakers in response to health-conscious consumers [11]. 

Due to increased demand for authentic and traditional foods, the reintroduction of ancient wheat and old wheat genotypes in bakery products is gaining a lot of interest [12,13,14]. Bread is one of the oldest staple foods worldwide, and it comes in different forms owing to its high versatility of ingredients and in the bread-making process [15,16,17]. Gluten is the main protein of wheat grain; it is composed of gliadins-conferring dough viscosity and extensibility and glutenin-conferring dough elasticity and strength [18,19]. Glutenins are also essential to hold the gas produced during the process of fermentation [20]. Protein percentage, gluten quantity, and composition, and the allelic composition and expression of gliadin and glutenin subunits, are in part responsible for the quality of the dough and final wheat end-product [4,21,22]. However, ancient wheats are known to have low technological quality, despite their high grain protein percentage due to their weak gluten index. 

Although *Triticum aestivium* is commonly used in making bread, South Italy has a long history of *Triticum durum* wheat-based bread. Nevertheless, Italian modern durum genotypes showed to be unsuitable for bread due to their unbalanced ratio of tenacity over extensibility [4]. Therefore, recent breeding programs in Italy worked on the developing of improved wheat using ancient grains by either a selection from landraces and local populations, as in the case of einkorn genotypes Monlis and Norberto (which used to be known as ID331), which were claimed to be suitable for bread-making or even by crossing emmer with durum wheat (*Triticum durum* Desf.) genotypes, as in the case of Padre Pio and Giovanni Paolo [7,23,24]. These latter were claimed to be suitable for pasta- making [25,26]. These new genotypes were created with the aim to improve their technological features and their adaptability to different environments and resistance to diseases and lodging in the marginal lands of central and southern Italy [27]. In this light, the objective of the present work was to evaluate the suitability of these improved einkorn and emmer genotypes for bread-making. This study followed a holistic approach by assessing wheat flour composition and dough rheological properties using different techniques and finally bread quality to enable an in-depth characterization of the bread-making ability of these genotypes. Regarding the studied set, all of the varieties are suitable for organic farming systems. Monlis, Norberto, Giovanni Paolo, and Padre Pio are new varieties with an improved grain yield and have improved agronomic and qualitative traits. In particular, they have good resistance to lodging and to major fungal diseases. They also have an improved protein composition and technological features suiting bread-making and baking goods and pasta [23,28,29]. Khorasan and Cappelli have been shown to have good baking properties [19,30]. Garfagnana is an Italian emmer landrace and certified IGP (Indication of Geographic protection). It is highly productive among emmer cultivars. 

## 2. Materials and Methods

### 2.1. Wheat Genotypes

A total of seven genotypes cultivated under the same environmental conditions in Ottava (Sardinia, Italy, 41° N; 8° E; 80 m above sea level) over two growing seasons were included in this study:

- One old durum wheat, *T. turgidum* subsp. *durum* Desf., Senatore Cappelli (Cappelli, selected 1914);
- Khorasan wheat, originated from Egypt and identified as *T. turgidum* subsp. *turanicum*;
- Two emmer genotypes, *T. turgidum* subsp. *dicoccum* shubler, Padre Pio (registered 2016) and Giovanni Paolo (registered 2008), selected by using the pedigree-selection method from the population obtained from a cross between the *T. dicoccum* schubler line and *T. turgidum* subsp. *durum* Desf;
- One emmer landrace, *T. turgidum* subsp. *dicoccum*, Garfagnana;
- Two einkorn genotypes of the *T. monocuccum* subsp. *monocuccum* genotypes, Monlis (registered 2006) and Norberto (registered 2017).

The raw materials of both seasons for each genotype were mixed and ground in an experimental laboratory of the university in Ottava using a stone miller and sifted to collect the resulting flours. 

### 2.2. Determination of Flour Properties

The flour nitrogen percentage was estimated using a Carbon/Hydrogen/Nitrogen Determinator (CHN 628 Series, Leco Corporation, St. Joseph, MI, USA). Each sample (80 mg) was added to a specific aluminum foil which was accurately folded and then placed inside the instrument’s sample holder. The combustion temperature was set at 1050 °C [31]. Before analysis, the instrument was calibrated against certified standards. Nitrogen data were used to calculate the protein percentage as N percentage × 5.7. Moisture, fat, and ash contents were calculated according to AACC Approved Methods of Analysis. Three determinations were performed for each sample. Total carbohydrates were obtained by subtracting the moisture, ash, lipid, and protein contents from 100. The moisture content (g/100 g flour) of the different varieties were as follows: Cappelli (12.4), Garfagnana (12.3), Giovanni Paolo (12.2), Kamut (11.9), Monlis (12.5), Norberto (12.1), and Padre Pio (12.0).

For protein characterization, albumins-globulins, gliadins, and glutenins were sequentially extracted, as previously described [19]. Total protein content was estimated by adding albumin-globulin, gliadin, and glutenin peaks. The relative percentage of each protein fraction, albumin and globulin, and gliadin and glutenin, were calculated from the total protein. Three determinations were performed for each sample. The gliadin over glutenin ratio was calculated by dividing the gliadin peak over the glutenin peak.

### 2.3. Determination of Dough Properties

Pasting properties and stirring number: Pasting curves and the stirring number (SN) were measured using a Rapid Visco Analyser (RVA), (Newport Scientific, Warriewood, Australia). The pasting temperature (°C), peak time (when peak viscosity occurred; min), peak viscosity (maximum hot paste viscosity), breakdown (peak viscosity minus holding strength or minimum hot paste viscosity), setback (final viscosity minus holding strength), and final viscosity (end of the test after cooling to 50 °C and holding at this temperature) were calculated as per AACC Method 76-21.02, while the stirring number was obtained by AACC Method 22-08.02. All the viscosity parameters are expressed in mPa s. Dough samples were prepared by dispersing 3.5 g of flour, in 25 mL of distilled water, into an aluminum canister. Doughs were then subjected to heating and cooling following these steps: holding at 50 °C for 1 min, gradual heating to 95 °C, holding at 95 °C for 2 min and 30 s, cooling to 50 °C, and lastly holding at 50 °C for 2 min. Three determinations were performed for each sample.

Starch damage: Starch damage was measured using Chopin SDmatic, and values were obtained by the AACC 76-33.01 Approved Method. Three determinations were performed for each sample.

Flour water absorption: Farinograph curves were obtained using a model 810105001 Brabender instrument (Brabender OHG, Duisburg, Germany). The water absorption, mixing time, degree of stability, and softening index (12 min after peak time) were measured according to AACC Approved Method 54-21.02. Three determinations were performed for each sample. Out of the output of this analysis, water absorption values were reported in the present study.

The rheology properties of the dough were analyzed using Alveograph, TA.XT analyzer and Rheometer. All analyses were performed in triplicate.

For Alveograph measurements, the strength of the dough (W), tenacity (P), extensibility (L), and the ratio of tenacity (P) to extensibility (L), were determined using a model MA87 Chopin alveograph (Group Tripette & Renaud, Villeneuve- La-Garenne, France). 

Resistance to extension (R) and extensibility (E) and stickiness was determined using a TA.XT2 plus, Stable Microsystems, Godalming, UK, equipped with a Kieffer dough and gluten extensibility rig. The analysis was performed following these settings: Measure Force in Tension; Data Acquisition Rate, 200 pps; Pre-test speed, 2.0 mm/s; Test speed, 3.3 mm/s; Post-test speed, 10.0 mm/s; Distance, 75.0 mm; Trigger force, Auto-5g. Doughs were placed in the rig and were allowed to rest for different resting times (RT: 45, 70, 90, and 135 min) under controlled environmental conditions (temperature, 22 ± 2 °C) [32]. For each resting time, at least four measurements were taken for each sample. R (g) and E (mm) were determined by recording the peak force and the distance at the extension limit, respectively [33], using Software Texture Expert Exceed, version 2.54, Godalming, UK. Dough stickiness was measured at room temperature using an SMS/Chen–Hoseney dough stickiness rig (A/DSC) and a 25 mm Perspex cylinder probe (P/25P) (Stable Micro-Systems, Surrey, UK). A millimeter of dough was extruded, relaxed for 30 s, and placed under a 25 mm cylindrical probe (probe SMS P/25). Dough viscosity (i.e., resistance to extension) was measured as the maximum positive force (N)”.

For rheometer measurements, doughs were subjected to small deformation rheological measurements using an MCR 92 rotational rheometer (Anton Paar GmbH, Inc., Graz, Austria) equipped with a Peltier-temperature-controlled system. The test was performed at 50 °C using a 60 mm serrated plate–plate geometry with a 2 mm gap between the plates. After lowering the upper plate, the excess sample was trimmed off, and a thin layer of silicon oil was used to cover the exposed surface to prevent moisture loss during the test. Prior to starting the test, the experimental gels were rested for 5 min between the plates to allow sample relaxation. A frequency sweep test was carried out over the range 0.1–10 Hz at 50 °C using a target strain of 0.01%, which fell within the linear viscoelastic region previously determined by running a strain sweep test at a constant frequency of 10 Hz and with a strain that varied over the range 0.001–100. Values of storage modulus (G′), loss modulus (G″), and loss tangent (tan δ) were recorded at a frequency of 1 Hz. 

Dough development and CO_2_ production during yeast activity: The maximum height reached by the dough (Hm) and the total volume of gas produced and retained during fermentation following the addition of yeast at 2% and salt at 1.8% to the flour and water were measured using a Chopin Rheo F4. Curves and values were obtained by the AACC 89-01.01 Approved Method. Three determinations were performed for each sample.

### 2.4. Bread-Making and Characterization

The dough was prepared by mixing flour (100%), water, salt (1.8%), and compressed yeast (2%) in a 10 Kg spiral mixer (Sigma srl, Brescia, Italy) for 10 min at low speed. The amount of water used to prepare each sample was calculated according to the Farinograph values. Bulk fermentation was carried out for 30 min at 30 °C and 85% relative humidity. The fermented doughs were then divided (500 g), molded into baking pans, placed in a proofer (30 °C, 80% relative humidity) until they rose to double their original size, and baked for 35 min at 230 °C in an electric oven (Europa, Molina di Malo; VI, Vicenza, Italy). After baking, the loaves were cooled at room temperature and analyzed after 2 h. Two productions were made for each formulation. For each production, all analyses were performed in triplicate.

Bread loaf volume was measured using the small seeds displacement method (AACC Standard 10-05.01). The specific volume was calculated as bread volume (mL) over bread weight (g). 

Bread mechanical properties (hardness, cohesiveness, springiness, and chewiness) were recorded in a TA.XT2 plus, Stable Microsystems, Godalming, UK using a 36-mm cylindrical probe (P36R), a speed rate of 1 mms^−1^, 40% penetration depth, and a gap of 30 s between compressions on three central slices (thickness 20 mm) of 1 loaf. Results were elaborated using Software Texture Expert Exceed, version 2.54.

The yellow color index (YI) of flour and bread crust and crumbs was determined using a model CR 300 colorimeter (Minolta, Osaka, Japan).

Statistical analysis: Analysis of variance (ANOVA) was performed at a significance level of α = 0.05. Significant differences among the mean values were calculated using Tukey’s test (*p* ≤ 0.05). Pearson correlation coefficients (r) were calculated to determine the associations between flour and bread properties. To summarize the relationships between products and breads, principal component analysis (PCA) was carried out. All experimental data were statistically analyzed using SPSS software (version 24.0; SPSS Inc., Chicago, IL, USA).

## 3. Results and Discussion

### 3.1. Flour Properties

Table 1 shows that the chemical composition of the wheat flours differed significantly. Monlis and Norberto had a protein content similar to Cappelli and Khorasan. The values of protein content were found within the same range of a study focusing on a set of old Italian durum [4] but were lower than the values found in a previous study focused on einkorn wheat [34]. This variability could be due to differences in the conditions of the environment [6,35]. The carbohydrate content was within the same range as the previous study [36,37]. The amylose content significantly varied among the studied genotypes in accordance with previous studies [10,37]. Giovanni Paolo had a high amylose percentage, which could contribute to dough strengthening and might result in breads with a dense texture [38,39]. The fat content was found within the range of variability of previous works [40]. The two einkorn genotypes had, considerably, the lowest ash content, while the emmer landrace (Garfagnana) had the highest amount of ash content consistent with a previous study [41]. 

Overall, high variability was observed among the studied genotypes in terms of the chemical composition underlining the genetic diversity among the studied set. Even though Giovanni Paolo and Padre Pio resulted from a cross using the same pedigrees and were cultivated in the same environment, they had different macronutrient contents. This highlights the importance of the use of ancient grains as a genetic reservoir to preserve biodiversity and to develop new grains with high nutritional quality. 

Regarding protein composition (Table 2), all of the genotypes had the same content of albumin and globulin except for Garfagnana. Our results of glutenin are lower than the ones found in the old, intermediate, and modern durum varieties of the previous study [42]. The discrepancy in the results could be associated with genetic (a different set of cultivars) and environmental (rainfall and temperature) differences [6]. Cappelli, Khorasan, and Padre Pio had a high gliadin content and a low glutenin content, leading to a high GLIGLU, while Giovanni Paolo and Garfagnana had a low gliadin content and high glutenin content, leading to a low GLIGLU. Interestingly, the emmer landrace (Garfagnana) and the two einkorn genotypes had a glutenin content higher than Cappelli and Khorasan. This can be useful in future breeding programs for selecting genotypes with high glutenin contents. The values of the GLIGLU are much higher than those found in Tunisian old durum wheat genotypes (0.4–1.16) [37] and lower than in old Italian durum wheat varieties [6]. TheGLIGLU ratio is directly associated with bread quality. This explains why genetic programs focused on integrating a better version of gluten fractions allelesin ancient grains, to improve the balance between glutenin and gliadin and, therefore, to reach an appropriate balance between viscosity and extensibility in the dough used for leavened bread-making [43].

### 3.2. Dough Properties

The stirring number varied significantly among the studied genotypes (Table 3). Giovanni Paolo had the lowest value of the stirring number, which could indicate a higher α-amylase activity [44]. Monlis, followed by Norberto and Khorasan, had the highest values. The low α-amylase activity of einkorn genotypes was previously reported [40]. In terms of starch damage (SD), the studied genotypes can be divided into three groups, which are: (i) high SD, attributed to Cappelli and Khorasan, (ii) low SD, attributed to einkorn genotypes (Monlis and Norberto), and (iii) intermediate SD, attributed to both the landrace and improved emmer genotypes. However, all our genotypes had an SD below 7.5%, which is considered the threshold for good baking quality for durum wheat [45]. 

According to the Farinograph results (Table 3), Giovanni Paolo had the highest water absorption value, which can be attributed to its hydrophilic nature. The einkorn genotypes (Norberto and Monlis) had the lowest values. The stability time varied significantly among the studied genotypes. Giovanni Paolo had the highest value (7.2 min), which is within the same range as rice and bean flours (9 and 8 min, respectively) [46]. The long development time of the dough was correlated to poor quality for baking, limited machinability, and relaxing stretchable properties [46]. Khorasan and Cappelli showed a medium stability time. The rest of the genotypes had values within the same range of soft wheat (1.6) [46].

Giovanni Paolo and Garfagnana had both the highest glutenin content and the lowest GLIGLU, two parameters strictly related to dough strength; however, they had completely different dough properties. Giovanni Paolo showed the highest tenacity to the extensibility ratio (P/L) and flour strength (W), while Garfagnana showed the lowest values of P/L and W. This confirms that the interaction of numerous parameters, e.g., grain composition, protein content, gluten components content, and genetic and environmental factors, are behind the quality of the wheat dough [4]. Except for Giovanni Paolo, the W values of the studied genotypes are in the same range as the old Italian durum varieties but lower than in modern Italian varieties [42]. The baking industry requires high W values (>180 10^−4^ J) combined with a balanced P/L index (0.40–0.50) [47]. Following these references, none of the studied genotypes are suitable for good bread-making performance. However, these references are set for common wheat doughs, and we believe that the references that range for durum wheat doughs should be different. 

The resistance (R) differed significantly among the genotypes and was found to be lower compared to soft wheat flour [48]. Cappelli and Giovanni Paolo have the highest R values, probably due to their high amounts of SD and water absorption since a strong correlation was found between these two factors (r = 0.78, *p* < 0.05, r = 0.97, and r < 0.01, respectively). In terms of the E, Giovanni Paolo significantly showed the highest value, which is within the same range of soft wheat flour with medium strength. The rest of the genotypes showed E values within the same range of weak soft wheat flour [48]. These differences might be related to their protein content. For stickiness, no trend was found in association with the history of breeding of the studied genotypes. 

Significant differences were observed among the studied genotypes in terms of pasting parameters (Table 3). Giovanni Paolo had the lowest values of peak and final viscosities, breakdown, and setback. The low starch viscosity properties of Giovanni Paolo could be due to its high-water absorption, which is confirmed by the negative correlation (r values ranging from −0.78 to −0.96 and *p* < 0.01) between the water absorption and the pasting properties (i.e., the peak and final viscosities, breakdown, and stirring number). The high amylose content of Giovanni Paolo might contribute to the low viscosity, but also plays an important role in the starch retrogradation rate [49]. Consequently, Giovanni Paolo could have the best bread shelf-life properties. High pasting properties were found to be negatively correlated to the dough P/L and W, which means that genotypes such as Norberto and Monlis are weak flours, and their resulting doughs recorded the highest viscous modulus (G″). Khorasan and Garfagnana had the highest setback, implying a high degree of retrogradation (Table 3). 

All of the dough samples corresponded to a predominant elastic nature behavior, G′  >  G″, in accordance with previous findings [50,51,52]. G′ and G″ showed a similar trend where Monlis and Norberto had the highest values (showing similar values, statistically) compared to the rest of the genotypes (which were statistically similar to each other). Damaged starch was found negatively correlated with G’ and G”, leading to firmer doughs. The low value of G” could be due to the existence of interactions between the proteins and the other components of the dough. Tan δ values were found ranging from 0.43 to 0.51, which is in the same range as those of soft wheat (0.44). 

Regarding the fermentation parameters (Table 4), Giovanni Paolo had the highest values of Hm and the total volume of CO_2_ produced and retained but the lowest coefficient of retention. While the einkorn genotypes (Monlis and Norberto) showed the opposite trend. High gas production capacity is associated with a higher content of total water-soluble sugars. In fact, a negative correlation was obtained between the stirring number and the produced and retained volume of CO_2_ (r = −0.82, *p* < 0.02 for both). Thus, the high dough development of Giovanni Paolo is related to its high amylase activity compared to the rest of the genotypes. 

### 3.3. Bread Properties

**Figure 1:**
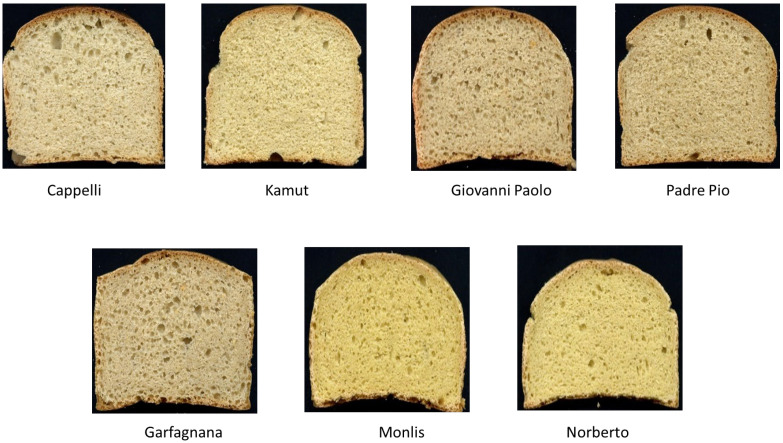
Pictures of the slices of the different breads.

Significant differences were found among the bread characteristics indicated in Table 5. The einkorn genotypes (Monlis and Norberto) had the highest yellow index of bread crust due to the high content of carotenoids [53], followed by the emmer landrace (Garfagnana), Khorasan, and the improved Giovanni Paolo and Padre Pio genotypes, while Cappelli had the lowest value (Figure 1, Table 5). These values were found to be higher than the yellow index of common wheat genotypes reported in a previous study due to a higher pigment content in durum wheat, einkorn, and emmer genotypes compared to common wheat varieties [53]. The yellow index of the breadcrumbs followed a similar trend to that of the crust color, which is also higher compared to that of common wheat crumbs [36]. 

Cappelli had the highest specific volume followed by Garfagnana and Padre Pio, while the einkorn genotypes had the lowest values. The bread-specific volumes are similar to those of a set of old Italian durum varieties but higher than those of a modern variety (Svevo) [4]. It was expected that Giovanni Paolo would show a high volume due to its Hm and CO_2_ production. However, its high W and P/L might have limited its volume increase. 

The results showed that the firmness of the breads was significantly (*p* ≤ 0.05) different as a function of genotype. A negative correlation between volume and crumb firmness (r = −0.76, *p* < 0.05) was obtained, which is consistent with previous results [54,55]. The einkorn genotypes had the highest values for bread firmness, while Cappelli and Giovanni Paolo were the softest. The einkorn genotypes also had the highest chewiness, which was double the value recorded by Giovanni Paolo. Hardness and chewiness were positively linked with peak viscosity (r = 0.81, *p* < 0.02 for both) and negatively linked with starch damage (r = −0.88, *p* < 0.009 and r = −0.85, *p* < 0.015) and the rheofermentometer measurements (r > 0.85, *p* < 0.015 for all parameters). As for cohesiveness, three groups can be identified: (i) Cappelli with the highest value, (ii) Khorasan, Padre Pio, Monlis, and Norberto having intermediate values, and (iii) Giovanni Paolo and Garfagnana having the lowest values. Cappelli showed the highest resilience followed by Khorasan and Padre Pio. Monlis and Norberto showed similar values, followed by Garfagnana, while Giovanni Paolo showed the lowest value. These results suggest a relevant impact of breeding on breadcrumb texture, but no trend was observed as a function of breeding history. 

### 3.4. Principal Components Analysis

**Figure 2:**
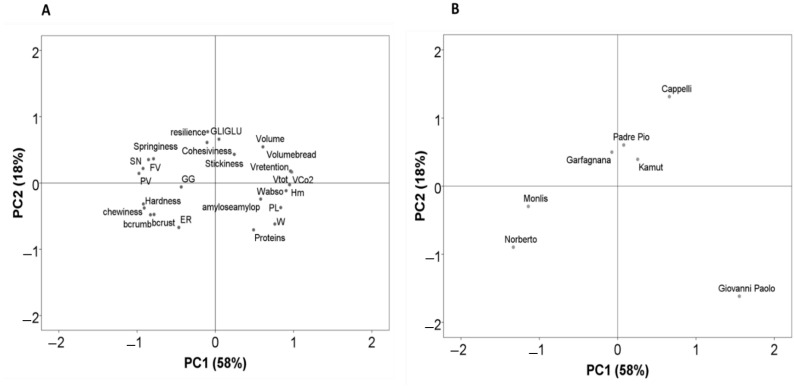
Biplot of flour, dough, and bread quality characteristics. (**A**): biplot of the first two components; (**B**): rotated principal scores of reformulated vegetable creams projected into the first two principal components. Legend: glutenin and gliadin percentage (GLU% and GLI%), peak viscosity, breakdown, setback, final viscosity, stirring number (SN), starch damage (SD), water absorption (water abso), maximum dough height (Hm), the total and retained volume of CO_2_ produced (V tot CO_2_ and V retained CO_2_), tenacity over elasticity ratio (P/L), Energy (W), bread-specific volume (Volume), hardness, springiness, cohesiveness, and resilience.

Figure 2A illustrates the first two axes explaining the 76% variation of a PCA based on flour, dough, and bread quality parameters of our seven studied genotypes. PC1 explained 58% of the total variability and was positively associated with bread volume, water absorption, and the rheofermentometer and alveograph parameters and negatively associated with the pasting parameters, springiness, hardness, and chewiness. PC2 explained 18% of the total variability and was positively correlated with GLIGLU, resilience, and cohesiveness. The contribution of ER and GG to the variability seen is less pronounced than the one of PL and W. By projecting the studied genotypes on the factorial space created by the first two principal components (Figure 2B), we were able to discriminate:

- Cappelli was characterized by good fermentation properties and a high bread volume, confirming previous results [4];
- Garfargnana, Padre Pio, and Khorasan were clustered in the middle, and this reflects high similarities among the genotypes, mostly in resilience, cohesiveness, and stickiness;
- Monlis and Norberto, which shared the same gluten subunits profile, were found on the same side opposing the other genotypes and were characterized by high chewiness, hardness, and high viscosity, and this could be due to their genetic characteristics being a diploid, having only the AA genome, and lacking the HMW and LMW glutenin subunits, B1 and B3, which were proved to play a key role in the quality of wheat end products [19];
- The emmer genotype, Giovanni Paolo, was discriminated against due to its high protein content and rheological features, i.e., a P, E, and W reflecting the high tenacity of the dough, which explains the low resilience of the final bread despite its good loaf volume and low firmness.

Overall, high biodiversity in the features was found among the studied set. Considering the breeding history, the einkorn genotypes had high similarities, but the two emmer genotypes (Padre Pio and Giovanni Paolo) were found to be significantly different in terms of their gluten subunits, grain composition, dough rheology, and bread quality.

## 4. Conclusions

Ancient and old wheat varieties were used for the selection of new, improved genotypes to preserve biodiversity and, at the same time, because of their ability to grow in marginal areas and to produce bread with acceptable quality. The different grain characteristics of the studied wheat genotypes showed a significant impact on dough pasting, fermentation, and bread properties. Owing to its dough tenacity and strength, Giovanni Paolo could be a dual-purpose variety suitable for pasta and for bread-making. Padre Pio could imitate the bread made by Khorasan, usually appreciated by consumers. The einkorn genotypes, Monlis and Norberto, are not suitable for bread fermentation but, instead, could be further explored for their suitability in making flatbreads. These results suggest a high versatility in improved einkorn and emmer wheat features that can be explored to make different types of breads in response to consumers’ demand for traditional, authentic, and nutritious breads and highlight the importance of exploring the genetic biodiversity of ancient and old wheat varieties for the development of new wheat varieties with improved characteristics suitable in the food industry.

## Figures and Tables

**Table 1:** Chemical composition of improved and ancient Italian genotypes.

Genotypes	Protein Content (g/100 g Flour)	Carbohydrates (g/100 g Flour)	Fat (g/100 g Flour)	Ash (g/100 g Flour)	Amylose/Amylopectin	Amylose (g/100 g of Starch)
Cappelli	14.2 ^b^	70.8 ^d^	1.38 ^c^	1.25 ^a^	0.38 ^ab^	27.5 ^ab^
Khorasan	13.4 ^c^	72.5 ^a^	1.24 ^c^	1.04 ^c^	0.42 ^a^	29.4 ^a^
Giovanni Paolo	16.6 ^a^	68.8 ^e^	1.25 ^c^	1.14 ^b^	0.44 ^a^	30.6 ^a^
Padre Pio	13.6 ^c^	71.7 ^b^	1.54 ^b^	1.17 ^b^	0.40 ^ab^	28.4 ^ab^
Garfagnana	12.4 ^d^	72.8 ^a^	1.25 ^c^	1.28 ^a^	0.33 ^b^	24.6 ^b^
Monlis	13.5 ^c^	71.4 ^bc^	1.83 ^a^	0.79 ^d^	0.38 ^ab^	27.4 ^ab^
Norberto	14.6 ^b^	71.0 ^cd^	1.58 ^b^	0.71 ^e^	0.37 ^ab^	26.7 ^ab^

Means with the same lowercase letter are not statistically different by Tukey’s test for *p* < 0.05.

**Table 2:** Protein composition of improved and ancient Italian genotypes.

Genotypes	Albumin and Globulin (%)	Gliadin (%)	Glutenin (%)	GLI/GLU (Dimensionless)
Cappelli	24.6 ^a^	57.3 ^a^	18.1 ^bc^	3.21 ^ab^
Khorasan	24.3 ^a^	58.3 ^a^	17.4 ^c^	3.37 ^a^
Giovanni Paolo	20.3 ^a^	45.5 ^b^	34.2 ^a^	1.74 ^c^
Padre Pio	25.4 ^a^	57.9 ^a^	16.7 ^c^	3.78 ^a^
Garfagnana	16.4 ^b^	53.6 ^ab^	30.0 ^a^	1.79 ^c^
Monlis	22.8 ^a^	51.5 ^ab^	25.7 ^b^	2.32 ^b^
Norberto	24.1 ^a^	48.4 ^b^	27.5 ^b^	2.23 ^b^

Means with the same lowercase letter are not statistically different by Tukey’s test for *p* < 0.05.

**Table 3:** Dough properties of improved and ancient Italian genotypes.

**Genotypes**	**Stirring Number (RVU)**	**Starch Damage (SD, UCD)**	**Water Absorption (%)**	**Stability (min)**	**P/L**	**Dough Strength (W, 104 J)**	**Resistance (R, g)**	**Extensibility (E, mm)**
Cappelli	158 ^d^	3.26 ^a^	64.1 ^b^	2.6	2.23 ^b^	84 ^c^	17.3 ^a^	21.6 ^b^
Khorasan	199 ^b^	3.26 ^a^	61.4 ^c^	5.9	1.68 ^bc^	91.3 ^b^	13.6 ^b^	24.4 ^b^
Giovanni Paolo	110 ^e^	2.59 ^b^	67.7 ^a^	7.2	3.76 ^a^	302.2 ^a^	17.0 ^a^	35.4 ^a^
Padre Pio	181 ^c^	2.25 ^b^	55.3 ^d^	1.5	1.25 ^bcd^	40.9 ^e^	10.9 ^c^	21.1 ^b^
Garfagnana	183 ^c^	2.23 ^b^	54.7 ^de^	1.4	0.53 ^d^	23.9 ^f^	9.20 ^d^	19.6 ^b^
Monlis	230 ^a^	1.57 ^c^	53.6 ^ef^	1.8	0.85 ^cd^	39.8 ^e^	9.20 ^d^	17.0 ^b^
Norberto	211 ^b^	1.18 ^c^	53.2 ^f^	1.8	0.66 ^cd^	47.0 ^d^	9.20 ^d^	25.2 ^b^
**Genotypes**	**Stickiness (N)**	**Peak Viscosity (mPa s)**	**Final viscosity (mPa s)**	**Breakdown (mPa·s)**	**Set Back (mPa·s)**	**Elastic modulus (G′, Pa)**	**Viscous modulus (G″, Pa)**	**Tan δ (G′/G″)**
Cappelli	0.48 ^cd^	1740 ^d^	2219 ^d^	665 ^c^	1144 ^c^	22,856 ^b^	9986 ^b^	0.44 ^ab^
Khorasan	0.49 ^bc^	2195 ^c^	2966 ^b^	662 ^c^	1433 ^a^	22,066 ^b^	9984 ^b^	0.45 ^ab^
Giovanni Paolo	0.47 ^d^	986 ^e^	1223 ^e^	456 ^d^	693 ^d^	17,531 ^b^	7575 ^b^	0.44 ^ab^
Padre Pio	0.48 ^cd^	2219 ^c^	2620 ^c^	913 ^b^	1313 ^b^	12,403 ^b^	6013 ^b^	0.49 ^ab^
Garfagnana	0.59 ^a^	2504 ^b^	2981 ^b^	953 ^b^	1430 ^a^	21,048 ^b^	9925 ^b^	0.47 ^ab^
Monlis	0.50 ^b^	3035 ^a^	2975 ^b^	1134 ^a^	1156 ^c^	60,824 ^a^	25,992 ^a^	0.43 ^b^
Norberto	0.38 ^e^	2990 ^a^	3195 ^a^	950 ^b^	1156 ^c^	52,844 ^a^	26,884 ^a^	0.5 ^a^

Means with the same lowercase letter are not statistically different by Tukey’s test for *p* < 0.05.

**Table 4:** Fermentation properties of the seven studied genotypes.

Genotypes	Maximum Development Reached by the Dough (Hm, mm)	Total Volume of Gas Given off in mL (V tot CO_2_, mL)	Total Volume Retained CO_2_ (mL)	Coefficient of Retained CO_2_ (%)
Cappelli	52.7 ^b^	1547 ^b^	1315 ^b^	85.0 ^bc^
Khorasan	48.8 ^bc^	1530 ^b^	1310 ^b^	85.6 ^bc^
Giovanni Paolo	58.2 ^a^	1716 ^a^	1434 ^a^	83.6 ^c^
Padre Pio	45.9 ^cd^	1476 ^b^	1276 ^c^	86.4 ^bc^
Garfagnana	50.0 ^bc^	1456 ^b^	1276 ^b^	87.6 ^b^
Monlis	37.5 ^e^	1090 ^c^	1007 ^b^	92.4 ^a^
Norberto	42.2 ^de^	979 ^c^	936 ^c^	95.7 ^a^

Means with the same lowercase letter are not statistically different by Tukey’s test for *p* < 0.05.

**Table 5:** Bread properties of the seven studied genotypes.

Genotype	Yellow Index of Crust	Yellow Index of Crumb	Volume (mL/g)	Firmness (N)	Springiness	Cohesiveness	Chewiness (N)	Resilience
Cappelli	20.6 ^d^	17.5 ^g^	2.82 ^a^	17.6 ^c^	0.96 ^ab^	0.82 ^a^	13.8 ^cd^	0.45 ^a^
Khorasan	29.3 ^b^	22.5 ^c^	2.43 ^cd^	20.0 ^b^	0.94 ^bc^	0.79 ^b^	14.8 ^bc^	0.41 ^b^
Giovanni Paolo	26.9 ^c^	20.4 ^d^	2.58 ^bc^	18.0 ^c^	0.92 ^c^	0.76 ^c^	12.6 ^d^	0.36 ^e^
Padre Pio	28.9 ^bc^	19.3 ^e^	2.63 ^ab^	20.1 ^b^	0.94 ^bc^	0.79 ^b^	14.9 ^bc^	0.40 ^bc^
Garfagnana	29.9 ^b^	18.2 ^f^	2.75 ^ab^	21.2 ^b^	0.95 ^ab^	0.76 ^c^	15.3 ^b^	0.38 ^d^
Monlis	37.3 ^a^	32.7 ^a^	2.29 ^d^	33.0 ^a^	0.97 ^a^	0.78 ^b^	25.0 ^a^	0.40 ^c^
Norberto	36.8 ^a^	30.4 ^b^	2.37 ^d^	34.6 ^a^	0.96 ^ab^	0.79 ^b^	26.1 ^a^	0.40 ^c^

Means with the same lowercase letter are not statistically different by Tukey’s test for *p* < 0.05.

## Data Availability

Not applicable.

## References

[1] Boukid F., Folloni S., Sforza S., Vittadini E., Prandi B. (2018). Current Trends in Ancient Grains-Based Foodstuffs: Insights into Nutritional Aspects and Technological Applications. Compr. Rev. Food Sci. Food Saf..

[2] Mefleh M. (2021). Cereals of the Mediterranean Region: Their Origin, Breeding History and Grain Quality Traits. Cereal-Based Foodstuffs: The Backbone of Mediterranean Cuisine.

[3] Boukid F. (2021). Cereal-Based Foodstuffs: The Backbone of Mediterranean Cuisine.

[4] Mefleh M., Conte P., Fadda C., Giunta F., Piga A., Hassoun G., Motzo R. (2019). From Ancient to Old and Modern Durum Wheat Varieties: Interaction among Cultivar Traits, Management, and Technological Quality. J. Sci. Food Agric..

[5] Lafiandra D., Sestili F., Sissons M., Kiszonas A., Morris C.F. (2022). Increasing the Versatility of Durum Wheat through Modifications of Protein and Starch Composition and Grain Hardness. Foods.

[6] Mefleh M., Motzo R., Samson M.F., Morel M.H., Giunta F. (2020). N Partitioning between Gluten Fractions in a Set of Italian Durum Wheat Cultivars: Role of the Grain N Content. Foods.

[7] Cadeddu F., Motzo R., Mureddu F., Giunta F. (2021). Effects of Clipping on the Nitrogen Economy of Four Triticum Species Grown in a Mediterranean Environment. Field Crops Res..

[8] Boukid F., Dall’Asta M., Bresciani L., Mena P., Del Rio D., Calani L., Sayar R., Seo Y.W., Yacoubi I., Mejri M. (2019). Phenolic Profile and Antioxidant Capacity of Landraces, Old and Modern Tunisian Durum Wheat. Eur. Food Res. Technol..

[9] Shewry P.R., Hey S. (2015). Do “Ancient” Wheat Species Differ from Modern Bread Wheat in Their Contents of Bioactive Components?. J. Cereal Sci..

[10] Yacoubi I., Kharrat N., Boukid F., Khanfir E., Hamdi K., Sayar R., Yong S. (2022). Durum Wheat Breeding before and after 1970 in Tunisia: Changes in Yield Components and Quality Attributes. BAOJ Biotechnol..

[11] Șerban L.R., Păucean A., Man S.M., Chiş M.S., Mureşan V. (2021). Ancient Wheat Species: Biochemical Profile and Impact on Sourdough Bread Characteristics—A Review. Processes.

[12] Boukid F., Gentilucci V., Vittadini E., De Montis A., Rosta R., Bosi S., Dinelli G., Carini E. (2020). Rediscovering Bread Quality of “Old” Italian Wheat (*Triticum Aestivum* L. Ssp. Aestivum.) through an Integrated Approach: Physicochemical Evaluation and Consumers’ Perception. LWT.

[13] Giunta F., Pruneddu G., Zuddas M., Motzo R. (2019). Bread and Durum Wheat: Intra- and Inter-Specific Variation in Grain Yield and Protein Concentration of Modern Italian Cultivars. Eur. J. Agron..

[14] Ruisi P., Ingraffia R., Urso V., Giambalvo D., Alfonzo A., Corona O., Settanni L., Frenda A.S. (2021). Influence of Grain Quality, Semolinas and Baker’s Yeast on Bread Made from Old Landraces and Modern Genotypes of Sicilian Durum Wheat. Food Res. Int..

[15] Boukid F. (2022). Flatbread—A Canvas for Innovation: A Review. Appl. Food Res..

[16] Boukid F., Rosell C.M. (2022). The Nutritional Quality of Wholegrain and Multigrain Breads Is Not Necessarily Better than White Breads: The Case of Gluten-Free and Gluten-Containing Breads. Int. J. Food Sci. Nutr..

[17] Pasqualone A., Caponio F., Pagani M.A., Summo C., Paradiso V.M. (2019). Effect of Salt Reduction on Quality and Acceptability of Durum Wheat Bread. Food Chem..

[18] Boukid F., Mejri M., Pellegrini N., Sforza S., Prandi B. (2017). How Looking for Celiac-Safe Wheat Can Influence Its Technological Properties. Compr. Rev. Food Sci. Food Saf..

[19] Mefleh M., Conte P., Fadda C., Giunta F., Motzo R. (2020). From Seed to Bread: Variation in Quality in a Set of Old Durum Wheat Cultivars. J. Sci. Food Agric..

[20] Dhaka V., Khatkar B.S. (2015). Effects of Gliadin/Glutenin and HMW-GS/LMW-GS Ratio on Dough Rheological Properties and Bread-Making Potential of Wheat Varieties. J. Food Qual..

[21] Dexter J.E., Matsuo R.R. (1977). Influence of Protein Content on Some Durum Wheat Quality Parameters. Can. J. Plant Sci..

[22] Padalino L., Mastromatteo M., Lecce L., Spinelli S., Conte A., Alessandro Del Nobile M. (2015). Optimization and Characterization of Gluten-Free Spaghetti Enriched with Chickpea Flour. Int. J. Food Sci. Nutr..

[23] De Vita P., Nicosia O.L.D., Nigro F., Platani C., Riefolo C., Di Fonzo N., Cattivelli L. (2007). Breeding Progress in Morpho-Physiological, Agronomical and Qualitative Traits of Durum Wheat Cultivars Released in Italy during the 20th Century. Eur. J. Agron..

[24] Giunta F., Pruneddu G., Motzo R. (2019). Grain Yield and Grain Protein of Old and Modern Durum Wheat Cultivars Grown under Different Cropping Systems. Field Crops Res..

[25] Brandolini A., Hidalgo A., Plizzari L. (2010). Storage-Induced Changes in Einkorn (*Triticum Monococcum* L.) and Breadwheat (*Triticum Aestivum* L. Ssp. Aestivum). Flours. J. Cereal Sci..

[26] Pasini G., Greco F., Cremonini M.A., Brandolini A., Consonni R., Gussoni M. (2015). Structural and Nutritional Properties of Pasta from *Triticum Monococcum* and *Triticum Durum* Species. A Combined ^1^H NMR, MRI, and Digestibility Study. J. Agric. Food Chem..

[27] Pagnotta M.A., Mondini L., Atallah M.F. (2005). Morphological and Molecular Characterization of Italian Emmer Wheat Accessions. Euphytica.

[28] De Vita P., Riefolo C., Codianni P., Cattivelli L., Fares C. (2006). Agronomic and Qualitative Traits of *T. Turgidum* Ssp. Dicoccum Genotypes Cultivated in Italy. Euphytica.

[29] Codianni G.G.E.P.N.D.F. (2000). Mosé e Padre Pio Due Nuovi Genotipi Di Farro (*Triticum Dicoccum* Schübler). Inf. Agrar..

[30] Valli V., Taccari A., Di Nunzio M., Danesi F., Bordoni A. (2018). Health Benefits of Ancient Grains. Comparison among Bread Made with Ancient, Heritage and Modern Grain Flours in Human Cultured Cells. Food Res. Int..

[31] Cannas M., Pulina S., Conte P., Del Caro A., Urgeghe P.P., Piga A., Fadda C. (2020). Effect of Substitution of Rice Flour with Quinoa Flour on the Chemical-Physical, Nutritional, Volatile and Sensory Parameters of Gluten-Free Ladyfinger Biscuits. Foods.

[32] Kieffer R., Garnreiter F., Belitz H.D. (1981). Beurteilung von Teigeigenschaften Durch Zugversuche Im Mikromaßstab. Z. Lebensm.-Unters. Forsch..

[33] Collar C., Andreu P., Martínez J.C., Armero E. (1999). Optimization of Hydrocolloid Addition to Improve Wheat Bread Dough Functionality: A Response Surface Methodology Study. Food Hydrocoll..

[34] Hidalgo A., Brandolini A. (2014). Nutritional Properties of Einkorn Wheat (*Triticum Monococcum* L.). J. Sci. Food Agric..

[35] Giunta F., Motzo R., Fois G., Bacciu P. (2015). Developmental Ideotype in the Context of the Dual-Purpose Use of Triticale, Barley and Durum Wheat. Ann. Appl. Biol..

[36] Senay S., Bilge B., Catherine S.S., Ovando-Martinez M. (2019). Starch Digestibility Properties of Bread from Hard Red Spring Wheat Cultivars Released in the Last 100 Years. Cereal Chem..

[37] Boukid F., Vittadini E., Prandi B., Mattarozzi M., Marchini M., Sforza S., Sayar R., Seo Y.W., Yacoubi I., Mejri M. (2018). Insights into a Century of Breeding of Durum Wheat in Tunisia: The Properties of Flours and Starches Isolated from Landraces, Old and Modern Genotypes. LWT.

[38] Park C.S., Baik B.K. (2007). Characteristics of French Bread Baked from Wheat Flours of Reduced Starch Amylose Content. Cereal Chem..

[39] Li C., Dhital S., Gidley M.J. (2022). High-Amylose Wheat Bread with Reduced in Vitro Digestion Rate and Enhanced Resistant Starch Content. Food Hydrocoll..

[40] Hidalgo A., Brandolini A., Ratti S. (2009). Influence of Genetic and Environmental Factors on Selected Nutritional Traits of *Triticum monococcum*. J. Agric. Food Chem..

[41] Zaharieva M., Ayana N.G., Al Hakimi A., Misra S.C., Monneveux P. (2010). Cultivated Emmer Wheat (*Triticum Dicoccon* Schrank), an Old Crop with Promising Future: A Review. Genet. Resour. Crops Evol..

[42] Giunta F., Bassu S., Mefleh M., Motzo R. (2020). Is the Technological Quality of Old Durum Wheat Cultivars Superior to That of Modern Ones When Exposed to Moderately High Temperatures during Grain Filling?. Foods.

[43] Barak S., Mudgil D., Khatkar B.S. (2013). Relationship of Gliadin and Glutenin Proteins with Dough Rheology, Flour Pasting and Bread Making Performance of Wheat Varieties. LWT–Food Sci. Technol..

[44] Newberry M., Zwart A.B., Whan A., Mieog J.C., Sun M., Leyne E., Pritchard J., Daneri-Castro S.N., Ibrahim K., Diepeveen D. (2018). Does Late Maturity Alpha-Amylase Impact Wheat Baking Quality?. Front. Plant Sci..

[45] Sissons M., Ames N., Egan N., Rhymer C. (2008). A Comparison of Two Instrumental Techniques Used to Discriminate the Cooking Quality of Spaghetti. Int. J. Food Sci. Technol..

[46] Fetouhi A., Benatallah L., Nawrocka A., Szymańska-Chargot M., Bouasla A., Tomczyńska-Mleko M., Zidoune M.N., Sujak A. (2019). Investigation of Viscoelastic Behaviour of Rice-Field Bean Gluten-Free Dough Using the Biophysical Characterization of Proteins and Starch: A FT-IR Study. J. Food Sci. Technol..

[47] Spaggiari M., Marchini M., Calani L., Dodi R., Di Pede G., Dall’asta M., Scazzina F., Barbieri A., Righetti L., Folloni S. (2022). Evolutionary Wheat Populations in High-Quality Breadmaking as a Tool to Preserve Agri-Food Biodiversity. Foods.

[48] Bardini G., Boukid F., Carini E., Curti E., Pizzigalli E., Vittadini E. (2018). Enhancing Dough-Making Rheological Performance of Wheat Flour by Transglutaminase and Vital Gluten Supplementation. LWT.

[49] Balet S., Guelpa A., Fox G., Manley M. (2019). Rapid Visco Analyser (RVA) as a Tool for Measuring Starch-Related Physiochemical Properties in Cereals: A Review. Food Anal. Methods.

[50] Makni M., Chtourou Y., Fetoui H., Garoui E.M., Boudawara T., Zeghal N. (2011). Evaluation of the Antioxidant, Anti-Inflammatory and Hepatoprotective Properties of Vanillin in Carbon Tetrachloride-Treated Rats. Eur. J. Pharmacol..

[51] Al-Khalifa H., Givens D.I., Rymer C., Yaqoob P. (2012). Effect of N-3 Fatty Acids on Immune Function in Broiler Chickens. Poult. Sci..

[52] Coţovanu I., Mironeasa S. (2022). Effects of Molecular Characteristics and Microstructure of Amaranth Particle Sizes on Dough Rheology and Wheat Bread Characteristics. Sci. Rep..

[53] Lomolino G., Morari F., Dal Ferro N., Vincenzi S., Pasini G. (2017). Investigating the Einkorn (*Triticum Monococcum*) and Common Wheat (*Triticum Aestivum*) Bread Crumb Structure with X-ray Microtomography: Effects on Rheological and Sensory Properties. Int. J. Food Sci. Technol..

[54] Cardone G., Rumler R., Speranza S., Marti A., Schönlechner R. (2021). Sprouting Time Affects Sorghum (Sorghum Bicolor [L.] Moench) Functionality and Bread-Baking Performance. Foods.

[55] Jekle M., Fuchs A., Becker T. (2018). A Normalized Texture Profile Analysis Approach to Evaluate Firming Kinetics of Bread Crumbs Independent from Its Initial Texture. J. Cereal Sci..

